# Low Back Pain Prevalence and Associated Factors in Iranian Population: Findings from the National Health Survey

**DOI:** 10.1155/2012/653060

**Published:** 2012-09-11

**Authors:** Akbar Biglarian, Behjat Seifi, Enayatollah Bakhshi, Kazem Mohammad, Mehdi Rahgozar, Masoud Karimlou, Sara Serahati

**Affiliations:** ^1^Pediatric Neurorehabilitation Research Center, University of Social Welfare and Rehabilitation Sciences, Tehran, Iran; ^2^Department of Physiology, Medicine School, Tehran University of Medical Sciences, Tehran, Iran; ^3^Department of Biostatistics, University of Social Welfare and Rehabilitation Sciences, Tehran, Iran; ^4^Department of Biostatistics, School of Public Health and Institute of Public Health Research, Tehran University of Medical Sciences, Tehran, Iran; ^5^Student Research Committee, University of Social Welfare and Rehabilitation Sciences, Tehran, Iran

## Abstract

*Background*. There are very few studies that had a sample size sufficient to explore the association between factors related to low back pain in a representative sample of the Iranian population. *Objective*. To examine the relationship between sociodemographic factors, smoking, obesity, and low back pain in Iranian people. *Methods*. We used Iranian adults respondents (*n* = 25307) from the National Health Survey. Adjusted odds ratios and 95% confidence intervals were estimated by using logistic regression. *Results*. The prevalence of low back pain was found in 29.3% of the studied sample. High age, female sex, being married, obesity, low-economic index, being smoker, in a rural residence, and low educational attainment, all increased the odds of low back pain. *Conclusions*. Our findings add to the evidence on the importance of obesity in relation to low back pain. These results can be used as a basis to reinforce health programs to prevent obesity.

## 1. Introduction

Low back pain (LBP) is a common medical problem [1, 2] that has many outcomes including disability [3] and taking time off from work [3, 4]. LBP is a major public health problem in the USA because more than 34 million (17%) adults reported LBP only, and 19 million (9%) reported LBP and neck pain in a 3 months duration [1]. One study in Canada estimated that 84% of adults have had LBP during their lifetime [5]. Average prevalences were 59% in UK [6], 70% in Denmark [7], and 75% in Finland [8]. In the general population, the prevalence of low back pain in a 1-month and annual duration ranges from 30% to 40% and 25% to 60%, respectively [9–11].

Overweight and obesity are also public health problems, due to their rapid growth in recent decades and their related health disorders, such as cardiovascular diseases, diabetes, some cancers, and other diseases [12]. In recent years, the statistics about obesity were appalling. In 2010, almost 43 million children (35 million in developing countries and 8 million in developed countries) were estimated to be overweight or obese [13]. It has been estimated that by 2020, type 2 diabetes and cardiovascular disease will account for almost 75% of all deaths worldwide [14].

Because of multifactorial nature of LBP, researchers have focused on both medical and nonmedical factors such as sociodemographic factors [15–18]. One potential predictor could be age. The positive association between age and LBP has been found in some studies [19, 20]. Another predictor is sex which has shown that LBP is more common in female than in male [2, 10, 21–24]. Bener et al. found a statistically significant association between place of residence and LBP among patients attending primary health care [19]. Some studies have shown that smoking is consistently associated with LBP [7, 25–28]. Findings from some studies showed that people with low levels of educational and low income have had the higher prevalence of LBP [3, 26–29].

Although obesity is a significant factor that is frequently associated with the presence of LBP [1, 30–32], but this association has not been confirmed by other studies [33, 34]. In a meta-analysis, Shiri et al. [24]. reported that obesity increases the risk of LBP [24]. Their findings showed that this association is stronger women than men.

Until now very few studies of the association between factors related to LBP have been carried out in a representative sample of Iranian population. Therefore, it is clear that there is a need to determine the factors related to LBP of people in this country. The present study was designed to assess relationships between age, sex, education level, place of residence, smoking, marital status, obesity, economic index, and active workforce and LBP among Iranian men and women. 

## 2. Materials and Methods

### 2.1. Data Set Examined

The National Health Survey in Iran (NHSI) is a survey designed to gain comprehensive knowledge and information about health care problems and difficulties in Iran. All necessary information for the conduct of this study was obtained from the NHSI database. These data were collected by the National Research Center of Medical Sciences and are presented partially at the Department of Biostatistics and Epidemiology, Tehran University of Medical Sciences for research. For the present study, pregnant women were excluded from the analyses, and the analyzed data included 25307 women and men aged 20–65 years. This study was approved by the Ethic Committee of the University of Social Welfare and Rehabilitation Sciences.

### 2.2. Study Outcome

Low back pain was defined as a binary variable with “yes” if a respondent has had low back trouble during the past 30 days.

### 2.3. Personal Status Covariates

**Obesity**

Height was measured in centimeters to the nearest 5 mm in a standing position, with shoes removed, using a wall-mounted stadiometer. Weight was measured to the nearest 0.1 kg with the subject in light indoor clothes, with shoes removed and emptied pockets. BMI (body mass index) was calculated as weight in kilograms divided by height in meters squared, and subjects were stratified into obese (BMI ≥ 30 kg/m^2^) and nonobese (BMI < 30 kg/m^2^).

**Education Level**

The respondents were categorized into three groups: those with low (0–8 years), moderate (9–12 years), or high (more than 12 years) education.

**Smoking**

Smoking status was dichotomized into smoker versus nonsmoker.

**Economic Index**

Due to ethical considerations, we did not ask respondents about their income, because they were afraid of paying their taxes. We surrogated economic index for their household income. Economic index was defined as square meter of living place divided by number of household.

**Demographic Variables**

Information about the respondent's age was based on their self-reported birth year (age); females versus males based on (sex); married versus single based on (marital status); rural versus urban based on (place of residence).

### 2.4. Data Analysis

Descriptive statistics were presented in terms of prevalence of each covariate between subjects with and without LBP. We used *χ* ^2^ tests to test significance of associations of each covariate with LBP. We applied logistic regressions to assess the association between obesity and LBP controlling for other covariates. In doing so, we first assessed a “crude” association between obesity and LBP without controlling for any variable. We then assessed the obesity-LBP relationship after controlling for all other covariates. No statistical interactions between different covariates were detected. The results are presented as odds ratios and their 95% confidence intervals (CI). The Hosmer-Lemeshow test was used in this model to evaluate the significance of improved port with introduction of additional variables. All analyses were carried out by using the SPSS software package, version 15.

## 3. Results 

Our data included 25307 women and men aged 20–65 years with a mean age of 36.19 year. The presence of LBP was found in 29.3% of the respondents. The mean economic index was 23.62 m^2^. Of the respondents, 12.6% were obese, 56.2% were women and 34.4% were rural. Altogether, 14.1% and 80.6% were smoker and married, respectively. Overall, 25.5% and 8.2% of respondents were classified as moderate and high educational levels, respectively.

The prevalence of each covariate across the LBP categories is presented in Table 1. Results of *χ* ^2^ tests showed that LBP was significantly associated with each of the covariates. 

We started by fitting a preliminary logistic model including only obesity and LBP to observe the influence of the potential confounders on LBP. This model showed that unadjusted LBP odds ratio was 1.62 (95% CI : 1.50–1.75). 

In logistic model controlling for age, sex, economic index, education level, marital status, place of residence, and smoking status, adjusted LBP odds ratio was 1.15 (95% CI :  1.06–1.24) for obesity. Comparing two models, we found that the LBP odds ratio decreased after adjustment for confounding variables, reducing by 47% from that of primary model. The results of the multivariate logistic model are shown in Table 2. 

In the multivariate analysis, the presence of LBP was associated with the female gender. The LBP odds ratio was 3.05 (95% CI : 2.84–3.27).

An association was observed between place of residence and LBP. The LBP odds ratio was 1.24 (95% CI : 1.17–1.32) for rural participants.

 We found a statistically significant association between smoking and LBP. For smoker participants, the adjusted odds ratio was 1.40 (95% CI : 1.27–1.53).

An association observed between marital status and LBP. The LBP odds ratio was 1.51 (95% CI : 1.39–1.64) for married.

Overall, subjects with higher education appeared as an associated factors with OR less than 1. Using basic education as the reference group, LBP odds ratios were 0.79 (95% CI : 0.73–0.85) and 0.65 (95% CI : 0.57–0.74) for the moderate and high groups, respectively. 

The Odds ratio of presence of LBP was inversely associated with the economic index. The LBP odds ratio was 0.996 (95 percent CI : 0.994–0.998). A 1-m^2^ increase in economic index has 1% decrease in the odds of LBP.

The odds of presence of LBP increased with age. The LBP odds ratio was 1.03 (95 percent CI : 1.029–1.034). We infer that a 1-year increase in age has 3% increase in the odds of LBP. 

## 4. Discussion

In this cross-sectional study, we assessed associations between the some factors and presence of LBP in men and women. In the first model (without confounders), unadjusted LBP ratio was 1.62 (95% CI : 1.50–1.75). Furthermore, we adjusted the odds ratio for common known covariates for LBP, for example, smoking and sociodemographic factors. After adjustment for confounding variables, obesity is positively associated with presence of LBP in adults.

Obesity behaved as an important predictor in two models, in agreement with the findings in the literature [30-32,1, 24]. Shiri et al. have reported that obesity is a risk factor for LBP in both cross-sectional and cohort studies [24]. Biomechanic and metabolic factors have been suggested to explain this relation. Obesity may cause LBP through metabolic syndrome. it is also possible that obesity and low back pain would be linked more directly via inflammatory mechanisms [35]. One study showed that people with high BMI increased risk of injury as well as higher injury-related expenditure [36]. Obesity has been shown as a risk factor for disc degeneration [37] and may increase the prevalence of LBP from this way. Because of a worldwide increase in the prevalence of obesity [14], it is reasonable to assume that the prevalence of back pain will continue to increase.

Studies have shown that the prevalence of LBP in the general population was higher in women than in men, and these findings are in agreement with the results of the present sample [2, 10, 21–24]. The sex difference could be related to gonadal steroid hormones such as estradiol and testosterone modulate sensitivity to pain and analgesia [38]. It is possible that LBP would have more influence on the life style habits in females than in males. Other variables such as diet, parity, and use of contraceptives may be relevant. 

Rural people had higher prevalence of LBP than urban people. We conclude that the geographical variation in prevalence of LBP in Iran is largely due to differences in propensity to consult a doctor once a symptom is present, and patient behaviour once symptoms have developed. Our results are consistent with some studies that reported the regional differences in the prevalence of LBP [29, 39].

In agreement with earlier findings [7, 25, 26, 28], our results showed that smoking, consistently associated with LBP. The association between smoking and LBP may be explained by the analgesic properties of nicotine [40]. Smokers might have stopped smoking on doctors' orders due to some disease that could be related to pain [41]. Smoking can effect on disc height [42]. Some biologically plausible explanations could be related to the effect of smoking on nutrition of the disc [43]. A positive correlation between severe disk degeneration and LBP was found in some studies [44]. 

Compared with unmarried, significantly increased odds of LBP were seen in married participants. Our results are consistent with some studies [45]. It is possible that the presence of a spouse may operate as a social factor on lack of LBP. It may include physiological mechanisms after their marriage.

According to the literature, people with lower educational attainment and economic index have an increased prevalence of LBP [3, 26–29]. These findings were also apparent among our participants. 

There are likely to be other aspects of environments and lifestyle which influence the presence of LBP.

It is possible that LBP is more likely to be reported by those with lower economic index and lower educational qualification. Higher education and economy may provide knowledge or resource thet influences on the lack of LBP.

Our findings suggest that age was an important associated factor revealing that prevalence of LBP increases with the increase of age. Age has also been a strong predictor for LBP in some previous studies [19, 20], possibly due to increasing degeneration of the tendons resulting from aging. 

There are several limitations in this study. The analysis is cross-sectional and therefore unable to infer causation. We did not examine all potentially important variables. Marital status could be categorized into legally married and nonmarried only. Nonmarried people are a very heterogeneous group and should be more closely examined in further studies. The lack of approach to the functional impairment is a limitation to this study. The chronic pain is also not included in our investigation. Although we cannot be certain of the temporal relationship between these variables and LBP, any of these can influence and limit the inferences about factors associated with LBP in this sample.

Despite these limitations, the NHS sampling design permits the representative sampling of households in Iran. The adult respondents included in this paper are therefore a valid representative sample of the Iranian population ages 20 years and older. Height and weight were actually measured rather than self-reported. It is well known that self-reports underestimate the prevalence of obesity.

## 5. Conclusion

Our findings add to the evidence on the importance of obesity in relation to low back pain. If confirmed in other studies, these findings will have implications with respect to attempts in preventing obesity in the population.

## Figures and Tables

**Table 1:** Descriptive prevalence of low back pain across study variable levels.

Variable	Low back pain		*P* value
	No.	%
Obesity			
Nonobese	5209	28.0	
Obese	1234	38.6	<0.001
Sex			
Men	2162	18.3	
Women	5409	37.5	<0.001
Place of residence			
Urban	4609	26.8	
Rural	2962	32.6	<0.001
Smoking			
Smoker	958	25.3	
Nonsmoker	6611	29.4	<0.001
Marital status			
Single	990	19.1	
Married	6581	31.2	<0.001
Education level			
Basic	5898	33.9	
Moderate	1342	20.2	<0.001
High	324	15.0	

**Table 2:** Adjusted^a^ odds ratios for low back pain among 25307 Iranian adults, National Health Survey in Iran, in the logistic analysis.

variable	OR^b^	95% CI^c^
Obesity		
Nonobese	1.00	
Obese	1.15	1.06–1.24
Sex		
Men	1.00	
Women	3.05	2.84–3.27
Place of residence		
Urban	1.00	
Rural	1.24	1.17–1.32
Smoking		
Nonsmoker	1.00	
Smoker	1.40	1.27–1.53
Marital status		
Single	1.00	
Married	1.51	1.39–1.64
Education level		
Basic	1.00	
Moderate	0.79	0.73–0.85
High	0.65	0.57–0.74
Economy index	0.996	0.994–0.998
Age	1.03	1.029–1.034

^a^ Adjusted for all other variables in the table.

^b^Odds ratio.

^c^Confidence interval.
